# Service readiness of primary healthcare facilities for dengue management in Bagmati Province, Nepal: a mixed method study

**DOI:** 10.1093/pubmed/fdaf079

**Published:** 2025-07-07

**Authors:** Sagar Parajuli, Hari P Kaphle, Nand R Gahatraj, Sunita Poudel, Arjun Poudel, Kusumsheela Bhatta, Gokarna Dahal

**Affiliations:** School of Health and Allied Sciences, Pokhara University, Pokhara-30, Kaski, Gandaki Province-Nepal; School of Health and Allied Sciences, Pokhara University, Pokhara-30, Kaski, Gandaki Province-Nepal; School of Health and Allied Sciences, Pokhara University, Pokhara-30, Kaski, Gandaki Province-Nepal; School of Health and Allied Sciences, Pokhara University, Pokhara-30, Kaski, Gandaki Province-Nepal; School of Health and Allied Sciences, Pokhara University, Pokhara-30, Kaski, Gandaki Province-Nepal; Patan Academy of Health Sciences, Lalitpur, Bagmati Province-Nepal; Epidemiology & Disease Control Division, Ministry of Health & Population Nepal, Kathmandu, Bagmati Province Nepal

**Keywords:** dengue, Nepal, primary healthcare facilities, service readiness

## Abstract

**Background:**

In Nepal, frequent dengue outbreaks have been reported in the past decades leading abrupt and substantial burden to healthcare system. Thus, this study aimed to assess dengue service readiness and factors influencing readiness at health facilities level.

**Methods:**

A convergent parallel mixed method study was conducted in 131 primary healthcare facilities of the Bagmati Province. Key informant interviews were carried out for exploring facilitators and barriers for dengue service readiness.

**Results:**

The majority of primary healthcare facilities were found with sub-optimal readiness for dengue i.e. 63.4% of facilities and readiness was found varied by health facility type, settings, ecological region, and districts. In the study, health facility type [Primary healthcare centers Adjusted Odds Ratio (AOR): 6.1, CI: 1.5–23.9], review and trend analysis practice [AOR: 3.6, CI: 1.1–11.6], and facilities meetings [AOR: 5.8, CI: 1.2–26.7] were identified as key predictors for service readiness. External supervision, quality assurance practice and stakeholder sensitization were explored as facilitators for the readiness.

**Conclusions:**

Improving and expanding dengue services in all primary healthcare facilities, and ecological regions, having regular facilities meeting, review and trend analysis practice and stakeholder sensitization can enhance dengue service readiness at facility level.

## Introduction

Dengue is emerging as ‘public health threat’ throughout the globe in the form of acute to large-scale epidemics with severe morbidity and mortality.^1^^,^^2^ The burden of dengue globally increased significantly by ˃300% since 2009.^3^ Today 3.9 billion people living at risk of dengue in 128 countries, among them dengue endemic reported in ˃100 countries.^3^^,^^4^

In Nepal, first dengue case was reported in 2005 and till 2006 no indigenous cases were reported.^5^ Dengue cases are now reported across all provinces and further, frequent outbreaks reported in the year 2010, 2013, 2016, 2019, and 2022 leading abrupt and substantial burden to healthcare system.^6–8^ The dengue case incidence was found alarming with an increment by five times more in 2018 compared to 2016 and caseload found increased drastically over the year.^7^^,^^9^ Similarly, shift in epidemiology was reported to upper hilly regions since 2016 and spatiotemporal epidemiology anticipated the future growing epidemics of dengue in Nepal due to climate change.^9^^,^^10^

In Nepal, endemicity and overall burden of disease was attributed to the capacity of health system to respond.^9^ In regard with health system readiness, several studies indicated low priority of dengue program at sub-national level, limited diagnostic capacities, lack of trained human resources, and lack of efficient disease surveillance as major reasons for frequent outbreaks of dengue in Nepal.^11–13^ Thus, the study aimed to assess dengue service readiness at primary healthcare facilities, which serves as the ‘first point of contact’ of people with the health system.

## Methods

### Study design

A convergent parallel mixed method study was conducted in 131 primary healthcare facilities in six randomly selected districts of the Bagmati Province as the province accounted for 78.2% of dengue cases and 68.4% of fatalities in 2022.^8^^,^^14^ A mixed method approach using convergent model was adopted in the study considering several evidences of using mixed method approach as the best for assessing service readiness in primary healthcare facilities and further convergent model was carried out to compare and relate quantitative results and qualitative findings.^15–18^ For qualitative study, ten key informant interviews (KIIs) were carried out for exploring facilitators and barriers for dengue service readiness.

### Sampling design

The sample size for the study was determined in accordance with WHO Service Availability Readiness Assessment (SARA) Implementation Guideline considering, margin of error-10%, Z-1.96, and proportion of service readiness-0.5.^19^ A stratified simple random sampling was used for the selection of facilities, where stratification was done by type of facility. For qualitative study, judgmental sampling was used for the selection of key informants with diverse experiences and perspectives based on working experience, and familiarity with programs. Data were collected from March–May 2024 using interview schedule with questionnaire, extensively based on WHO SARA Tool.

### Measurement of service readiness

Service readiness of facilities for dengue was measured by five domains, including basic amenities, staff and guideline, infection prevention and control, diagnostic capacity and essential medicines and commodities. Tracer items in each domain were finalized with experts including senior national dengue program administrator, tropical disease expert, public health researcher, entomologist, and health service provider to ensure relevance of items in the context of Nepal. Tracer items having content validity ratio of 1 and 0.6 in Lawshe method after experts’ consultation were only included. The availability of tracer item in each domain was measured as ‘binary variable’ (1 for Yes, 0 for No) and mean score for each domain was computed by dividing sum of scores by number of items and multiplied by 100. Each domain contributed equally to overall readiness score. A cut-off point of ‘70’ was determined in overall readiness score to categorize facilities into having optimal and suboptimal readiness taking reference of several studies.^20–23^ Facilities with 70 or ˃70 were considered having ‘optimal readiness’ in the study.

### Statistical analysis

Quantitative Data analysis was done using R V.4.3.3 and for qualitative analysis, Barun and Clark’s six-step thematic analysis with deductive coding was conducted based on the study’s conceptual framework. The process included familiarizing with the data, generating initial codes, and organizing them into the pre-determined themes outlined in the framework, followed by reviewing and refining themes. Bivariate analysis was performed to study association between variables and further multivariate analysis was carried out to identify predictors for the service readiness.

### Ethical considerations

The ethical approval for the study was provided by Nepal Health Research Council (Ref No. 1457/2024) and approval from Epidemiology and Disease Control Division (EDCD- Letter No:931/80/81), Health offices and local government was taken prior. Similarly, dates and time for data collection at facility was decided prior considering peak service hours to prevent interruption in service deliveries.

## Results & findings

### Health facility characteristics

Among 131 health facilities studied, 19.1% were basic hospitals, 9.9% primary healthcare centers (PHCC) and 71% were health posts (HP). More than half i.e. 66% of health facilities belonged to urban settings. Majority of health facilities were from hill (64.1%), followed by mountain (22.9%), and terai (13%). More than half (60.4%) facilities were found having monthly or more frequently meeting and 93.1% of health facilities were found having quality assurance practices (Table 1).

**Table 1:** Health facility characteristics.

HF Characteristics	Frequency (n = 131)	Percentage (%)
**Health Facility Type**		
Basic Hospital	25	19.1
Primary Healthcare Center	13	9.9
Health Post	93	71.0
**Health Facility Location: District**		
Bhaktapur	9	6.9
Chitwan	17	13.0
Dhading	22	16.8
Kathmandu	29	22.1
Sindhuli	24	18.3
Sindhupalchok	30	22.9
**Settings based on municipality**		
Urban	87	66.4
Rural	44	33.6
**Ecological regions**		
Mountain	30	22.9
Hill	84	64.1
Terai	17	13.0
**Distance to District Headquarters**		
Distance ≤ 10 KM	24	18.3
Distance > 10 KM	107	81.7
**Staff fulfillment status**		
Fulfilled as per sanctioned	52	39.7
Not fulfilled	79	60.3
**Contract Hiring at Health Facility**		
Yes	47	35.9
No	84	64.1
**Staff transfer in the last 6 months**		
Yes	35	26.7
No	96	73.3
**Months of In-charge in service**		
In position ≤ 12 Months	38	29
In position > 12 Months	93	71
**Frequency of health facility meeting**		
Monthly or more frequently	93	60.4
Once in every 2–3 months	23	14.9
Once in every 4–6 months	15	9.7
**Management committee meetings in last 4 months**		
Yes	47	35.9
No	84	64.1
**Observed availability of Health Facility Operation and Management Committee (HFOMC) Guideline**		
Observed	66	50.4
Reported not seen	41	31.3
Not available	24	18.3
**Availability of system for collecting client feedback & opinions**		
Yes	117	89.3
No	14	10.7
**Methods of client feedback and opinion collection** ^a^ *(Total responses = 160, n = 117)*		
Suggestion Box	113	86.3
Client Survey	3	2.3
Client Interview	8	6.1
Official Meeting	4	3.1
Informal discussion	32	24.4
**Lastly suggestions box open (n = 113)**		
Within 6 Months	27	23.9
6–12 Months	24	21.2
˃12 months	37	32.7
Not, opened yet	25	22.1
**Procedure of reviewing client feedback and opinions (n = 117)**		
Yes	90	76.9
No	27	20.6
**Quality Assurance Practice in Health Facility (n = 131)**		
Yes	122	93.1
No	5	3.8
Don’t know	4	3.1
**Observation of QA record (n = 122)**		
Observed-Records were well maintained	55	45.1
Reported, not seen	52	42.6
Observed- Records were not well maintained	15	12.3
**Minimum service assessment in the last fiscal year July 2022–June 2023**		
Conducted	120	91.6
Not conducted	11	8.4
**MSS Score Categories (n = 120)**		
˂50%	0	–
50%–70%	14	11.7
70%–85%	64	43.3
85%–100%	42	35

^a^Multiple responses.

**Figure 1:**
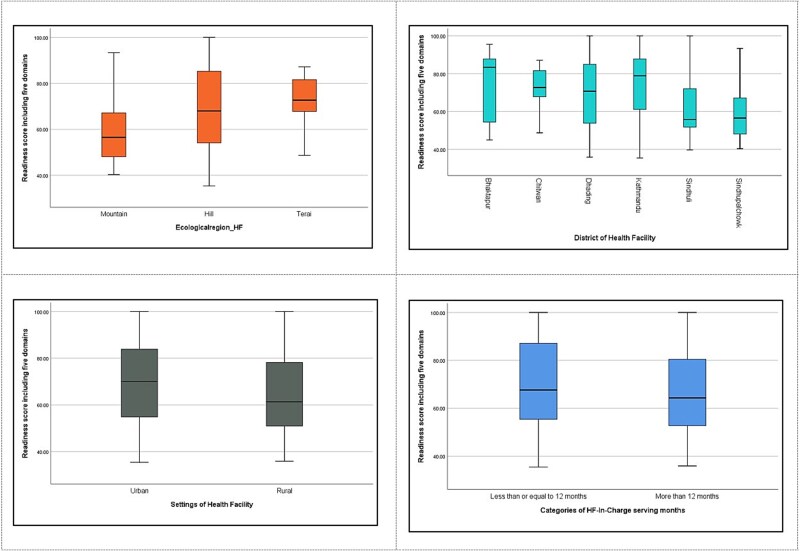
Box and whistler plot showing readiness score by health facility characteristics.

### Dengue service availability, disease burden, & health facility response

All health facilities were found performing clinical diagnosis of dengue. However, only half of health facilities i.e. 57.2% were reported with rapid diagnosis testing (RDT) service for dengue and among them, 69.3% reported availability of kits all times and 30.7% reported availability mostly during outbreak. More than half of facilities i.e. 54.2% reported supply of RDT kit by local government, and 9.9% reported self-purchasing of RDT kits whenever required. However, near to half of health facilities i.e. 45.3% reported stock out of kits in the past 6 months.

Among health facilities studied, more than one quarter had dengue cases ˃78, median of cases of two fiscal years. Regarding training, only 66.4% health facilities were found with service provider having training/orientation in dengue in the last 2 years and only 40.2% facilities were found with provider having training on case management. Further, half of health facilities i.e. 52.7% reported having training during outbreak only while 13% reported having training not available at all.

Likewise, only 28% health facilities were found with well-maintained specific register for dengue and among them 34.3% facilities found using register provided by Epidemiology and Disease Control Division. Majority of facilities (80.9%) were found reporting confirmed cases to local government while only one-quarter facilities (25.2%) were found having review and trend analysis practice. Likewise, in the last 12 months more than half (55.7%) health facilities had stakeholders’ orientation and 45% of facilities had search and destroy campaign in their catchment areas.

### Health facility readiness status

Among 131 health facilities studied, only 48 i.e. (36.6%) of facilities were found with optimal service readiness for the dengue. The average readiness score was found higher in primary healthcare centers (PHCC) (84.4, CI: 76.6–92.5), followed by basic hospital (83.5, CI, 78.5–88.5) and health post (HP) (59.7, CI, 56.5–62.9) (Table 2).

**Table 2:** Mean readiness score by domains and HF characteristics.

Variables	Basic Amenities	IPC	Staff & Guideline	Dengue Diagnosis	Essential Medicines & Commodity	Overall Readiness Score
**HF Type** *Mean, 95% Confidence Interval*						
Basic Hosp	93.7 (90.2–97.1)	93.3 (89.8–96.8)	56.0 (41–70.9)	84.0 (74.1–93.8)	90.6 (86.1–95.1)	83.5 (78.5–88.5)
PHCC	92.3 (84.7–99.8)	88.8 (79.7–97.9)	57.6 (33.5–81.8)	92.3 (80.9–100)	91.0 (84.3–97.6)	84.4 76.6–92.2)
HP	79.2 (75.9–82.5)	84.4 (81.9–86.9)	29.5 (22.5–36.5)	30.6 (22.6–38.6)	74.7 (71.3–78.0)	59.7 (56.5–62.9)
**Settings**						
Urban	85.2 (82.0–88.4)	85.8 (83.2–88.3)	39.1 (31.0–47.1)	51.7 (42.3–61.1)	80.6 (77.3–83.9)	68.5 (64.6–72.3)
Rural	79.5 (74.5–84.5)	88.1 (84.2–92.0)	34.1 (23.3–44.8)	37.5 (25.1–49.8)	76.8 (71.3–82.4)	63.2 (57.5–68.9)
**Ecological region**						
Mountain	79.9 (74.4–85.5)	85.5 (80.4–90.6)	28.3 (17.7–38.9)	21.6 (9.9–33.3)	72.7 (66.1–79.4)	57.6 (52.4–62.8)
Hill	84.6 (81.0–88.3)	88.2 (85.7–90.7)	39.2 (30.8–47.7)	51.1 (41.5–60.8)	80.1 (76.5–83.7)	68.7 (64.3–73.0)
Terai	82.3 (76.8–87.8)	80.3 (74.4–86.2)	44.1 (24.0–64.2)	70.5 (52.2–88.9)	87.2 (83.4–90.9)	72.9 (67.5–78.3)
**Districts**						
Bhaktapur	95.2 (89.7–100)	90.1 (84.9–95.2)	38.8 (13.2–64.5)	61.1 (23.7–98.4)	75.9 (60.1–91.7)	72.2 (57.1–87.3)
Chitwan	82.3 (76.8–87.8)	80.3 (74.4–86.2)	44.1 (24.0–64.2)	70.5 (52.2–88.8)	87.2 (83.4–90.9)	72.9 (67.5–78.3)
Dhading	84.4 (77.9–90.8)	86.8 (81.4–92.2)	38.6 (21.9–55.2)	54.5 (34.1–74.9)	80.2 (72.8–87.7)	68.9 (60.2–77.6)
Kathmandu	91.1 (86.9–95.3)	88.5 (83.5–93.4)	46.5 (30.5–62.5)	65.5 (50.1–80.8)	82.1 (75.6–88.7)	74.7 (67.7–81.8)
Sindhuli	73.2 (64.6–81.7)	88.4 (83.7–93.1)	31.2 (15.0–47.5)	27.0 (10.6–43.5)	79.1 (72.8–85.4)	59.8 (51.4–68.1)
Sindhupalc-hok	79.9 (74.4–85.5)	85.5 (80.4–90.6)	28.3 (17.7–38.9)	21.6 (9.9–33.3)	72.7 (66.1–79.4)	57.6 (52.4–62.8)
**Distance to district HQ**						
≤ 10 KM	89.2 (84.1–94.3)	86.1 (80.8–91.3)	43.7 (26.9–60.5)	58.3 (38.9–77.6)	76.3 (68.6–84.1)	70.7 (62.5–79.0)
> 10 KM	81.9 (78.8–85.0)	86.7 (84.3–89.0)	35.9 (29.0–42.9)	44.3 (36.2–52.5)	80.0 (76.9–83.1)	65.8 (62.3–69.2)
**Mean Score** **95% CI**	83.3 (80.5–86.0)	86.6 (84.4–88.7)	37.4 (31.0–43.7)	46.9 (39.4–54.4)	79.3 (76.5–82.2)	66.7 (63.5–69.9)

The overall mean readiness score was found varied by facility type, setting, and districts. The mean availability of items was found higher in domain infection prevention and control and lower in domain staff and guideline. Detailed information about availability of tracer items by each domain are provided in Supplementary File.

### Factors associated with service readiness and its predictors

In bivariate analysis, type of health facility, ecological regions, case load, reported outbreak in the district, stakeholder orientation, search and destroy campaigning, health facility meeting, management committee meetings, meeting focused on dengue, and minimum service standard (MSS) assessment were only found significant (Table 3).

**Table 3:** Factors associated with service readiness identified by bivariate analysis.

Variables	Facility Readiness n (%)		*χ*2, (df)	Unadjusted OR 95% CI	*P*-value
	Optimal 47(35.9)	Sub-optimal 84 (64.1)			
**Type of Health Facility**					**< 0.001** ^*^
Basic Hospital	19 (76%)	6 (24%)	31.8 (2)	8.2 (2.2–29.4)	**0.001** ^*^
PHCC	9 (69.2%)	4 (30.8%)		11.5 (4.0–32.7)	**< 0.001** ^*^
Health Post^RC^	20 (21.5%)	73 (78.5%)		1	
**Ecological Region**					**0.018** ^*^
Terai	8 (47.1%)	9 (52.9%)	9.21 (2)	5.7 (1.3–23.8)	**0.006** ^*^
Hill	36 (42.9%)	48 (57.1%)		4.8 (1.5–15.2)	**0.015** ^*^
Mountain^RC^	4 (13.3%)	26 (86.7%)		1	
**Distance to district HQ**					
Nearest ≤ 10 KM	12 (50%)	12 (50%)	2.2 (1)	1.9 (0.8–4.8)	0.133
Far > 10 KM^RC^	36 (33.6%)	71 (66.4%)		1	
**Staff fulfillment as per sanctioned positions**					
Fulfilled	14 (26.9%)	38 (73.1%)	3.5 (1)	2.0 (0.9–4.3)	0.09
Not fulfilled^RC^	34 (43.0%)	45 (57.0%)		1	
**HF-In-charge service duration in the facility**					
> 12 Months	32 (34.4%)	61 (65.6%)	0.6 (1)	0.7 (0.3–1.5)	0.407
≤ 12 Months^RC^	16 (42.1%)	22 (57.9%)		1	
**Case load in health facility (Median cases-78 in two FYs)**					
≥ 78 cases	26 70.3%)	11 (29.7%)	25.1 (1)	7.7 (3.3–18.1)	**< 0.001** ^*^
< 78 cases^RC^	22 (23.4%)	72 (76.6%)		1	
**Reported outbreak in the district**					
Reported last year	39 (50.6%)	38 (49.4%)	15.7 (1)	5.1 (2.2–11.9)	**< 0.001** ^*^
Not reported^RC^	9 (16.7%)	45 (83.3%)		1	
**Dengue Cases Review and Trend Analysis**					
In practice	23 (69.7%)	10 (30.3%)	20.7 (1)	6.7 (2.7–16)	**< 0.001** ^*^
Not in practice^RC^	25 (25.5%)	73 (74.5%)		1	
**Stakeholder orientation on dengue in last 12 months**					
Conducted	32 (47.9%)	38 (52.1%)	9.0 (1)	3.1 (1.4–6.8)	**0.013** ^*^
Not conducted^RC^	13 (22.2%)	45 (77.6%)		1	
Contd. **Search and destroy campaign in last 12 months**					
Conducted	32 (52.5%)	27 (45.8%)	14.3 (1)	4.1 (1.9–8.8)	**< 0.001** ^*^
Not conducted^RC^	16 (22.2%)	56 (77.8%)		1	
**External supervision in last 4 months**					
Yes	38 (44.2%)	48 (55.8%)	6.1 (1)	2.7 (1.2–6.3)	**0.013** ^*^
No^RC^	10 (22.2%)	36(77.8%)			
**Frequency of HF Meeting**					
Monthly or frequently	44 (47.3%)	49 (52.7%)	15.7 (2)	7.6 (2.5–23.2)	**< 0.001** ^*^
Once every 2–3 months^RC^	4 (10.5%)	34 (89.5%)	–		
**Management Committee Meeting in last 4 months**					
Had meeting	28 (59.6%)	19 (40.4%)	16.6 (1)	4.7 (2.1–10.1)	**< 0.001** ^*^
No meeting^RC^	20 (23.8%)	64 (76.2%)		1	
**Meeting focused on dengue in last year**					
Had meeting & action plan	22 (55.0%)	18 (45.0%)	8.3 (1)	3.0 (1.4–6.6)	**0.004** ^*^
No focused meeting^RC^	26 (28.6%)	65 (71.4%)		1	
**MSS Score categories**					
MSS Score ≥ 70	42 (42.9%)	56 (57.8%)	11.4 (2)	15.7 (2.0–121.8)	**< 0.001** ^*^
MSS Score < 70%^RC^	1 (4.5%)	21 (95.5%)		1	
**Quality Assurance practice**					
Observed and verified	16 (29.1%)	39 (70.9%)	1.5 (1)	0.5 (0.2–1.3)	0.158
Not observed^RC^	27 (41.5%)	38 (58.5%)		1	

^*^statistically significant at *p*-value < 0.05.

In multivariate logistic regression analysis, variables with *P*-value ˂0.25 in bivariate analysis and variance inflation factor (VIF) as numerical diagnostic for multicollinearity less than or equal to 2 were included. In multivariate analysis, type of health facility, frequency of health facility meeting and review and trend analysis practice were found statistically significant. Primary healthcare centers were found 6 times more likely to have dengue service readiness [ß coefficient: 1.81, AOR 95% CI:1.5–23.9]. In an overall, type of facility was identified as predictor for dengue service readiness however no statistically significant association was observed for basic hospital compared to health post. Likewise, health facilities having review and trend analysis were found 3.7 times more likely to have dengue service readiness [ß coefficient:1.29, 95% CI: 1.1–11.6]. Further, the odds of having optimal service readiness in health facilities with monthly or frequently meeting was found 5.8 times higher [ß coefficient:1.76, AOR 95% CI: 1.2–26.7] than facilities having not regular monthly meeting (Table 4).

**Table 4:** Predictors for dengue service readiness identified by multivariate analysis.

Variables	Unadjusted OR 95% CI	*P*-value	Adjusted OR 95% CI	*P*-value
**Type of Health Facility**		**< 0.001** ^*^		**0.032** ^*^
Basic Hospital	8.2 [2.2–29.4]	**0.001** ^*^	2.6 [0.4–15.9]	0.293
Primary Healthcare center	11.5 [4.0–32.7]	**< 0.001** ^*^	6.1 [1.5–23.9]	**0.008** ^*^
Health Post	1		1	
**Ecological region**		**0.018** ^*^		0.058
Terai	5.7 [1.3–23.8]	**0.006** ^*^	5.5 [1.2–24.8]	**0.025** ^*^
Hill	4.8 [1.5–15.2]	**0.015** ^*^	4.32 [0.36–51.2]	0.375
Mountain	1		1	
**Case load in the facility**				
≥ 78 cases in last two FYs	7.7 [3.3–18.1]	**< 0.001** ^*^	3.8 [0.8–17.1]	0.077
< 78 cases in two FYs	1			
**Review and Trend analysis**				
In practice	6.7 [2.7–16]	**< 0.001** ^*^	3.6 [1.1–11.6]	**0.028** ^*^
Not in Practice	1			
**External supervision in last 4 months**				
Yes, had supervision	2.7 [1.2–6.3]	**0.013** ^*^	1.7 [0.5–5.7]	0.345
No supervision	1		1	
**Frequency of HF Meetings**				
Monthly or more frequently	7.6 [2.5–23.2]	**< 0.001** ^*^	5.8 [1.2–26.7]	**0.022** ^*^
Not monthly mostly	1		1	
**Management Committee Meeting**				
Had in last 4 months	4.7 [2.1–10.1]	**<0.001** ^*^	0.8 [0.2–3.4]	0.853
No meeting in 4 months	1		1	
**Meeting focused on dengue in last year**				
Had meeting with action plan	3.0 [1.4–6.6]	**0.004** ^*^	0.6 [0.1–2.0]	0.435
No focused meeting	1		1	

^*^statistically significant at *p*-value < 0.05.

The multivariate regression model also fitted data with *P*-value ˂0.05 compared to null model. The accuracy of model was also reported 83.4% and area under the curve (AUC) was found 0.878 [95% CI 0.817–0.939]. The model also explained 36.64% variation in outcome variable i.e. dengue service readiness by type of facility, review and trend analysis practice and facility meeting.

### Facilitators and barriers of service readiness

Based on Guest *et al.* 2006 suggestions of minimum 12–20 interviews, initially 12 key informant interviews were planned for the study.^24^ However, simultaneous data analysis was carried out to ensure whether the ‘saturation point’ achieved or not and reaching data saturation at 9th interview and confirming with further one KIIs, sample size was concluded with 10 interviews consulting with supervisors.^25–27^

In qualitative study, readily availability of RDT kits at health facilities and training and orientation of health service providers since few years of outbreak was reported. Similarly, supply of RDT kits at health facilities by local government, practice of case reporting using different platform like social media, and multi-stakeholder sensitization on dengue were also explored which indicated readiness from municipality and health facility to some extent as expressed by key informants below.

#### Health facility characteristics

In health facility characteristics, staff availability and training on dengue was explored as facilitator for the service readiness while type of facility, health facility in proximity and distance to headquarters were identified as barriers.

‘Some health posts with labs from ….. municipality are also not provided with RDT kits, this may be due to being in proximity to tertiary healthcare facilities’- K01, Kathmandu.

#### Leadership and governance

Facilities having regular meeting, the practice of review and trend analysis, supervision and monitoring from higher level, local government support as well as leadership of management committee were found facilitating the service readiness as expressed by key informants.

‘In our facility, we review trend for most of diseases and make ourself preprepared for the future […..] in dengue last year we had 347 cases and based on that we made requisition for more than 500 RDT kits at very beginning, [……].’- K06, Dhading.

‘Last year, we had management committee meetings with an agenda on dengue as well and we developed action plan for search and destroy campaign. Also, availability of RDT kits was also the agenda, the chairperson himself coordinated with municipality immediately and ensured availability of kits.’ -K07, Bhaktapur.

‘Talking about monitoring, the upper level asks only about positive cases (laughs), apart from phone inquiries nothing else is done. Monitoring should be site visit, but it is only limited to phone call inquiry about number of cases’-K02, Kathmandu.

#### Perceived disease burden & facility response

However, perceived low disease risk and burden, and ineffective supervision & monitoring were identified as key barriers for dengue service readiness. Proactiveness of local government and facility for response taking initiatives were also acknowledged as key facilitator.

‘Now, we are having more dengue cases in the district, you can see the trend but still in the district we do have limited testing services, this may be due to district being looked as low risk being situated in mountain region.’-K04, Sindhupalchok.

‘[…….] (Showing messenger group of municipalities for case reporting), we do have a messenger group for reporting case of dengue by end of day to the local government, here you can see messages’-K05, Chitwan.

*Analyzing findings from both quantitative and qualitative study, some of convergent findings were identified including readiness of facility for dengue service delivery, leadership and governance factors influencing readiness however some of divergent findings were also depicted including management committee meetings, setting and proximity influencing readiness in qualitative study which wasn’t reflected in quantitative study.*

## Discussion

### Main findings of this study

Our study depicted majority of primary healthcare facilities with sub-optimal readiness for dengue service, whereas availability of tracer items for service readiness found varied by type of facility, settings, ecological region, and by districts as well. Only three in ten health facilities were found with optimal readiness for dengue services. Our study also revealed type of facility, frequency of facility meeting, as well as cases review and trend analysis practice in the facility as statistically significant key predictors for dengue service readiness. Further, external supervision, management committee meeting, review and trend analysis practice and staff fulfillment were also explored as facilitators for service readiness.

### What is already known on this topic

Despite growing epidemics of neglected tropical diseases including dengue throughout the globe, limited studies have been carried out in assessing primary healthcare facilities readiness and few studies carried out also revealed suboptimal readiness of health facilities for neglected tropical diseases. In Uganda only one quarter of health facilities were only found with malaria service readiness and the study of Sri Lanka also showed sub-optimal readiness for dengue service at primary healthcare facilities similar to our finding.^28^^,^^29^

Likewise, low prioritization of neglected tropical diseases in terms of training and services has been realized despite known burden globally.^30^^,^^31^ Several studies also identified limited trainings at national and sub-national level, inadequate trained staffs availability at health facilities and availability of training during only outbreak and peak season as key constraining factor for the dengue service readiness at facilities.^18^^,^^29^^,^^32^^,^^33^ These findings were also reflected in our study as lowest readiness score was found in domain staff and guideline.

Regarding service readiness, health facility characteristics including type, settings, ecological regions were found affecting service delivery and readiness in several studies conducted in Nepal including Nepal Health Facility Surveys.^28^^,^^34–39^ These findings also resembled with our study as dengue service readiness found varied by type of facility, settings and ecological regions.

### What this study adds

The study extends existing knowledge by providing more comprehensive and nuanced understanding of dengue service readiness at the healthcare facility level utilizing mixed method approach. Findings from both methods are well triangulated to provide best possible evidence for enhancing dengue service readiness as limited studies have been conducted using mixed method. Moreover, the service readiness of healthcare facilities in this study measured as a whole by five domains; basic amenities, infection prevention and control, dengue diagnostic capacity, staff and guideline and essential medicines and commodities. This provides comprehensive snapshot of health facilities readiness for dengue from essential basic amenities to service specific requirements.

The study also further advances understanding of the impact of leadership and governance factors as well as health facility characteristics on the service readiness for dengue. Looking into leadership and governance aspects, in our study health facilities having monthly meeting were found significantly associated with optimal service readiness which accords with several studies conducted in Nepal.^20^^,^^37^^,^^40^ Regular discussion on meeting for improving services and facility performance at facility level could the reason for higher readiness in facilities having regular meeting. Likewise, external supervision from higher level, management committee meetings, stakeholder sensitization and mobilization were found facilitating factor for enhancing service delivery and readiness in our study which are consistent with findings from countries including Tanzania, and Sudan.^20^^,^^40–42^

Further, the study also indulged into identifying factors associated with the service readiness for dengue and explored facilitators, barriers and reasons for suboptimal readiness through the qualitative study. Lack of dengue specific training on one side and less prioritization for the training throughout the year due to seasonal disease occurrence were identified as key factor influencing service readiness at the facility. Interestingly, frequent change in health facility leadership i.e. in-charge, and staff fulfillment status were also revealed as key factors influencing service readiness. The study also explored some initiatives from local government and health facilities for dengue prevention and control, including testing of every febrile case for dengue, scrub typhus and typhoid for early identification of dengue case and mobilization of Female Community Health Volunteers (FCHVs) and key stakeholders for search and destroy campaigns in the community.

### Limitations of the study

The study is believed to be of very first kind to study dengue service readiness in Nepal through mixed method, however quality aspects of services weren’t covered which comes under major limitation of the study. Similarly, the study used stratified simple random sampling for the selection of health facilities thus there could be some effect of stratification however expert consultation, and capacity mapping of facilities within and between strata were done priorly to the study.

The study was conducted in only 131 health facilities of the Bagmati Province, thus there could be limitation in the generalizability of the findings to wider scale due to small sample size. Likewise, the use of judgmental sampling for selection of key informants with diverse perspectives and experiences could be both the strength and limitations of the study as incorporating diverse perspectives will add value to the research but at the same time, there could be selection bias, as the selected participants may have unique experiences or perspectives not shared by others.

In this study, all key informants were health facility workers within the same province. Given the shared geographical context, thematic saturation was reached earlier than expected, as informants often reported similar experiences and challenges. However, we acknowledge that this focused sample might limit the generalizability of our findings to other provinces to other stakeholder groups.

### Conclusions

Enhancing service readiness for dengue at primary healthcare facilities seems very crucial in concern with increasing cases each year and frequent outbreaks in the country on one side and on another side for diversification in the healthcare approach and for reducing burden on tertiary healthcare facilities. Our study exploring factors influencing dengue service readiness identified prominent impact of leadership and governance factors along with health facility characteristics on the readiness. Thus, an integrated approach with consolidated efforts including stakeholder sensitization, effective monitoring and supervision from higher level, review and trend analysis practice in the facility as well as regular health facility meeting and meeting with key stakeholders could be encouraged for ensuring optimal dengue service readiness at facility level.

## Supplementary Material

Supplementary_File_Revised_(1)_fdaf079

## Data Availability

The dataset used and analyzed during the study are available from the corresponding author on reasonable request.

## Notes

**Sagar Parajuli,** MPH Graduate

**Hari P. Kaphle,** Associate Professor

**Nand R. Gahatraj,** Assistant Professor

**Sunita Poudel,** MPH Graduate

**Arjun Poudel,** MPH Graduate

**Kusumsheela Bhatta,** MPH Graduate

**Gokarna Dahal,** Public Health Administrator
