# Supplementary material for: Service readiness of primary healthcare facilities for dengue management in Bagmati Province, Nepal: a mixed method study
Source: J Public Health (Oxf). 2025 Jul 7;47(4):e640–51. doi: 10.1093/pubmed/fdaf079 (PMC12669995; doi:10.1093/pubmed/fdaf079)
Supplement: Supplementary_File_Revised_(1)_fdaf079 [file supplementary_file_revised_(1)_fdaf079.docx]

# Supplementary Files

**S1: Table showing distribution of tracer items by domains**

| **Domain and Tracer items** | **Frequency**  **(n=131)** | **Percentage (%)** |
| --- | --- | --- |
| **Basic Amenities** |  |  |
| Power | 115 | 87.8 |
| Availability of Improved water source | 128 | 97.7 |
| Auditory and visual privacy | 68 | 51.9 |
| Access to sanitation facility | 126 | 96.2 |
| Availability of communication device | 90 | 68.7 |
| Access to computer/laptop with internet | 121 | 92.4 |
| Emergency transportation availability | 119 | 90.8 |
| **Standard Precautions for Infection Prevention and Control** |  |  |
| Safe final disposal of sharp wastes | 117 | 89.3 |
| Safe final disposal of infectious wastes | 108 | 82.4 |
| Appropriate storage of sharp wastes | 117 | 89.3 |
| Appropriate storage of infectious waste | 121 | 92.4 |
| Availability of disinfectant | 111 | 84.7 |
| Availability of single use or AD syringe | 125 | 95.7 |
| Availability of soap & running water or hand rub | 126 | 96.2 |
| Availability of latex gloves | 121 | 92.4 |
| Availability of guidelines for standard precautions | 71 | 54.2 |
| Contd.. |  |  |
| **Dengue Diagnosis** |  |  |
| Availability of dengue diagnosis method (RDT) | 75 | 57.2 |
| Staff trained at dengue diagnosis | 48 | 36.6 |
| **Staff and Guideline** |  |  |
| Availability of National guideline in the facility | 63 | 48.1 |
| Staff trained in dengue case management | 35 | 26.7 |
| **Essential Medicines and Commodities** |  |  |
| Availability of at least one valid Paracetamol | 131 | 100 |
| Availability of at least one valid IV Fluid | 100 | 76.3 |
| Availability of at least one valid IV Cannula | 88 | 74.9 |
| Availability of at least one valid Pheniramine/Allergic | 88 | 67.2 |
| Availability of at least one valid ORS | 131 | 100 |
| Availability of at least one valid RDT Kits | 57 | 43.5 |

**S2: Table showing distribution of Dengue service availability in Health Facilities**

| **Dengue Service Availability** | **Frequency**  **(n)** | **Percentage (%)** |
| --- | --- | --- |
| **Availability of dengue diagnosis service** |  |  |
| Clinical diagnosis based on signs and symptoms | 131 | 100 |
| Availability of diagnosis service |  |  |
| RDT Test | 75 | 57.2 |
| Not available at all | 56 | 42.8 |
| Type of RDT kits available (n=75) |  |  |
| NS1 Ag + IgG and IgM Detection | 65 | 86.7 |
| NS1 Ag Detection | 10 | 13.3 |
| Availability of RDT kits (n=75) |  |  |
| Availability all times | 52 | 69.3 |
| Available during outbreak only | 23 | 30.7 |
| Source of availability of RDT kits* *(Total responses= 138, n=75)* | | |
| Supply by Local government | 71 | 54.2 |
| Supply by Provincial government | 23 | 17.6 |
| Supply by Federal government | 21 | 16.0 |
| Purchase by Health facility themselves | 13 | 9.9 |
| Support by EDPs | 10 | 7.6 |
| Stock out of RDT kits in past 6 months (n=75) |  |  |
| Yes | 34 | 45.3 |
| No | 41 | 54.7 |
| Case management service as identified in national guideline* *(Total responses=166, n=131)* |  |  |
| Group A Management service available | 131 | 100 |
| Group B Management service available | 35 | 26.7 |
| **Availability of Dengue related guideline** |  |  |
| National guideline on prevention, management and control of Dengue in Nepal, 2019 |  |  |
| Observed | 63 | 48.1 |
| Reported but not seen | 35 | 26.7 |
| Not available | 33 | 25.2 |

**S3: Table showing Dengue burden and Health Service Provider Training Status**

| **Dengue burden and Health Service Provider**  **Training Status** | | **Frequency**  **(n)** | **Percentage (%)** | | |
| --- | --- | --- | --- | --- | --- |
| Dengue cases reporting in last two fiscal years | |  |  | | |
| Yes | | 75 | 57.3 | | |
| No | | 56 | 42.7 | | |
| Reported cases in the FY 2078/79 Median = 19, Q1~ 15, Q3~49, Min.= 10, Max. = 1758 | | | | | |
| Reported cases in the FY 2079/80 Median = 48, Q1~ 33, Q3~85, Min.= 8, Max. = 1325 | | | | | |
| Case Load in the last two FYs Median = 78, Q1~ 53, Q3~126, Min. = 22, Max. = 3083 | | | | | |
| Case load in the facility taking median 78 as cut off | |  |  | | |
| Cases Reported ≤ 78 cases | | 94 | 71.8 | | |
| Cases Reported > 78 cases | | 37 | 28.2 | | |
| **Health Service Provider Training Status** | |  |  | | |
| HSPs with training/orientation in dengue in last 2 years | |  |  | | |
| Yes | | 87 | 66.4 | | |
| No | | 44 | 33.6 | | |
| HSPs with case management training (n=87) | |  |  | | |
| Yes | | 35 | 40.2 | | |
| No | | 52 | 59.8 | | |
| HSPs trained/oriented on any method-dengue diagnosis (n=87) | |  |  | | |
| Yes | | 48 | 55.1 | | |
| No | | 39 | 44.9 | | |
| HSPs trained/oriented with RDTs (n=48) | |  |  | | |
| Yes | | 45 | 93.8 | | |
| No | | 3 | 6.3 | | |
| Availability of dengue related trainings & orientations (n=131) | | | | | |
| Availability all times | | 45 | | | 34.4 |
| Available during outbreak only | | 69 | | | 52.6 |
| Not available at all | | 17 | | | 13.0 |

**S4: Themes and Sub-themes identified to explore status, facilitators and barriers**

|  | Themes | Sub-Themes | Supporting Verbatim |
| --- | --- | --- | --- |
| Status of Dengue Service Readiness | Service delivery at health facilities | Availability of dengue testing services at health facilities | *“Dengue has spread like epidemic in past, so the preparation was already in place from the municipality. Moreover, test kits are now available for more tests. Even the medicines are readily available.” - K01, Service Provider, Health Post, Hill* |
|  |  | Availability of management and referral services | *“[……] For dengue, you know all, it is management of symptoms so here, we provide first level of management for dengue like Paracetamol for fever management and refer cases to district hospital if there any chances of complications…..” – K07, ANM, Health Post, Hill* |
|  | Health Service Provider Availability and training | Health Service Providers Availability | *There is no specific person assigned as focal person for dengue program at our facility……” – K01, Lab Technician, Health Post, Hill* |
|  |  | Training & orientation of Health service providers | *“For dengue, till date to the best of my knowledge, there is no specific trainings. The rapid diagnostic testing training is available for malaria but not for dengue yet. Similarly, there is no specific case management training […].”-K03, Health Section Chief, Municipality, Sindhupalchok* |
|  | Recording and reporting for Dengue | Reporting to local government and use of DHIS 2 | *“[…….] (Showing messenger group of the municipality for dengue case reporting), we do have a messenger group for reporting case of dengue by end of day to the local government, here you can see messages”-K01, Lab Technician, Health Post, Kathmandu* |
|  |  | Use of lab register for dengue case recording | *“(Showing record register), we keep record of dengue tests here, this is for all lab tests. There is a register provided by Epidemiology and Disease Control Division, it is very long to fill and I am alone here to perform lab tests and maintain record both, so conveniently I use this register (showing record register maintained by the facility)”-K01, Lab Technician, Health Post, Kathmandu* |
|  | Essential Medicines & Commodities | Supply and stock of RDT kits | *“Talking about our PHC, the RDT Kits are not available enough for us to diagnose every fever case. There is uncertainty regarding supply and we have to test symptomatic patients. Apart from there, are unable to test every fever case.” -K02, In-charge, PHCCs, Kathmandu* |
|  |  | Availability of Essential Medicines & resources | *“We do have readily available Paracetamol as first line treatment, Dextrose glucose and anti-allergic medications; Avil whenever needed, that’s enough for dengue I guess (smiling) and if there further complication, we refer cases to […….]” K09, Basic Hospital, Bhaktapur* |
|  | Leadership and Governance | Preparedness from local government | *“Dengue has spread like a type of epidemic, so the preparation was already in place from the municipality. Moreover, test kits are now available for more tests. Even the medicines are readily available* […….….] *The preparation was already in place from the municipality. -K01, Service Provider, Health Post, Kathmandu* |
|  |  | Multi stakeholder Engagement | *“Now, there is budget from federal government for stakeholder orientation and ‘search and destroy campaign’. And our municipality have been providing orientation to multiple stakeholders including local representatives, FCHVs, municipal police and health workers and with support of them, search and destroy campaigning was done”-K06, Health Section Chief, Municipality, Chitwan* |
| Facilitators for the service readiness | Human resources | Staff availability & training on dengue | *“There is no such a focal person for dengue like for other programs, however lab staff Ms….in our facility is considered as the focal person for dengue, she coordinates and ensures availability of RDT kit, which has definitely helped in ensuring service readiness.”-K09, Hospital Chief, Basic Hospital, Bhaktapur* |
|  | Disease burden and response | Case load & outbreak in the district | *“In fiscal year 2079/80, we saw around 1800 cases. In this year, we have seen around 2800, 2700 cases. Due to that reason, in the health facilities having labs, we have managed them to be in a state of readiness by providing necessary kits.”-K08, Health Section Chief, Municipality, Dhading* |
|  |  | Trend Analysis practice in the facility | *“In our health facility, we review cases trend for most of diseases and make ourself preprepared for the future […..] in dengue last year we had 347 cases and based on that we made requisition for more than 500 RDT kits at very beginning, [……] in this way, we plan according to our review.”- K02, In-charge, PHCCs, Kathmandu* |
|  | Leadership and governance | Supervision and monitoring from higher level | *“Sometimes, it’s very guiding when we have supervision from higher level especially from Epidemiology and Disease control Division, it helps us to know our status and further provides us guidance and way forward”-K07, ANM, Health Post, Dhading* |
|  |  | Quality assurance practice in the facility | *“Every year, we do have minimum service standard assessment which helps us to know our status…. And identifies areas for improvement. Last year we had MSS of 96 and we were awarded by municipality, this motivates us to perform better […]”-K01, Lab Technician, Health Post, Kathmandu* |
|  |  | Facility meetings | *“In every 2-3 months, we conduct staff meeting where we discuss several aspects like health facility indicators status, administrative and management issues, as well as areas for improvement which ultimately support in better services delivery.”- K01, Lab Technician, Health Post, Kathmandu* |
|  |  | Coordination and Stakeholder engagement | *“We felt shortage in very first stage but in second phase fulfilled the shortage in collaboration with the government from FAIRMED Organization. And nowadays, we have sufficient kit [..]. -K04, Lab Technician, PHCCs, Sindhupalchok* |
|  |  | Local government support | *“We have been receiving various support from municipal level to ward level for dengue control. When the epidemic spread during the rainy season, we are been provided the orientation trainings for dengue control by municipality.”-K02, In-charge, PHCCs, Kathmandu* |
| Barriers for the service readiness | Health Facility characteristics | Type of health facility | *“Having laboratory services at primary health care centers and basic hospitals seems to be prioritized for dengue services, newly formed basic hospital 5 bedded are also provided with RDT kits for dengue testing however only few health posts are being provided, the RDT testing doesn’t require well maintained lab I guess, I don’t know why health posts are not being prioritized, we do have suspected cases but we can’t test them so we refer them to hospitals….[…]”-K07, ANM, Health Post, Dhading* |
|  |  | Near to district HQ & Higher HFs in proximity | *“[…………] hospital is quite near from here may be at the distance of 14 ...15/16 km so this may be the reason we are not provided with lab services. But yes, we are asking for it.”-K07, In-Charge, Health Post, Dhading* |
|  |  | Settings & Ecological region with perceived low risk | *“Now, we are having more dengue cases in the district, you can see the trend but still in the district we do have limited health facilities providing testing services, this may be due to district being looked as low risk being situated in mountain region.”-K04, Lab Technician, PHCCs, Sindhupalchok* |
|  |  | Lack of human resources and dengue specific trainings | *“Imagine 3 persons doing work of 5 persons, wouldn’t it compromise the quality of work thus staff fulfillment is very essential for delivery of any services, not only for dengue.” -K05, Medical Officer, Basic Hospital, Chitwan* |
|  | Disease burden & response | Perceived low disease risk | *“Following years, we can see increase in cases, and I guess few people only come here for testing as most of them go to Kathmandu being near from here…[…] the reported cases are only few numbers may be just 10-15% of total but based on cases reported, we see the district and the local government not actively and proactively leading dengue prevention and control program, this is due to perceived low risk, which is false.” -K04, Lab Technician, PHCCs Sindhpalchok* |
|  |  | Lack of timely response and action | *“The timely directive from federal and provincial government are also very essential for response and action, mostly we get to know about increasing cases through news rather than from our own health system, which results in delay in response and action…”- K09, In-charge, Basic Hospital, Bhaktapur* |
|  | Essential medicines and commodities | Shortage and stockout of RDT kits | *“We felt shortage of RDT Kits in very first stage, so we couldn’t test for dengue.” -K04, Lab Technician, PHCCs Sindhupalchok* |
|  |  | Gaps in supply and procurement | *“Previously, we used to have supply directly from district health office now kits are being supplied by local government, however there is supply from federal and provincial government, sometimes lack of coordination between them results into interruption in supply of kits and commodities necessary for the services.”-K01, Lab Technician, Health Post, Kathmandu* |
|  | Leadership and governance | Ineffective supervision & monitoring | *“Talking about monitoring, it only refers to asking questions by the upper level about how many positive cases are recorded (laughs). Apart from phone inquiries, nothing else is done yet. Monitoring should be site visit, inquiry about the management like if things are being done accordingly or not, but it is only limited to phone call inquiry about number of cases”-K02, In-charge, PHCCs, Kathmandu* |
|  |  | Limited stakeholder engagement | *“For effective implementation of ‘search and destroy’ campaign, multiple stakeholders should be oriented and mobilized, till today we do have practice of orienting local leaders only, but beyond them, it should focus on media, schools and local community groups like mother group.”- K06, Health Section Chief, Municipality, Chitwan* |

**S5: Spot map visualization of Health Facilities by Readiness Status**


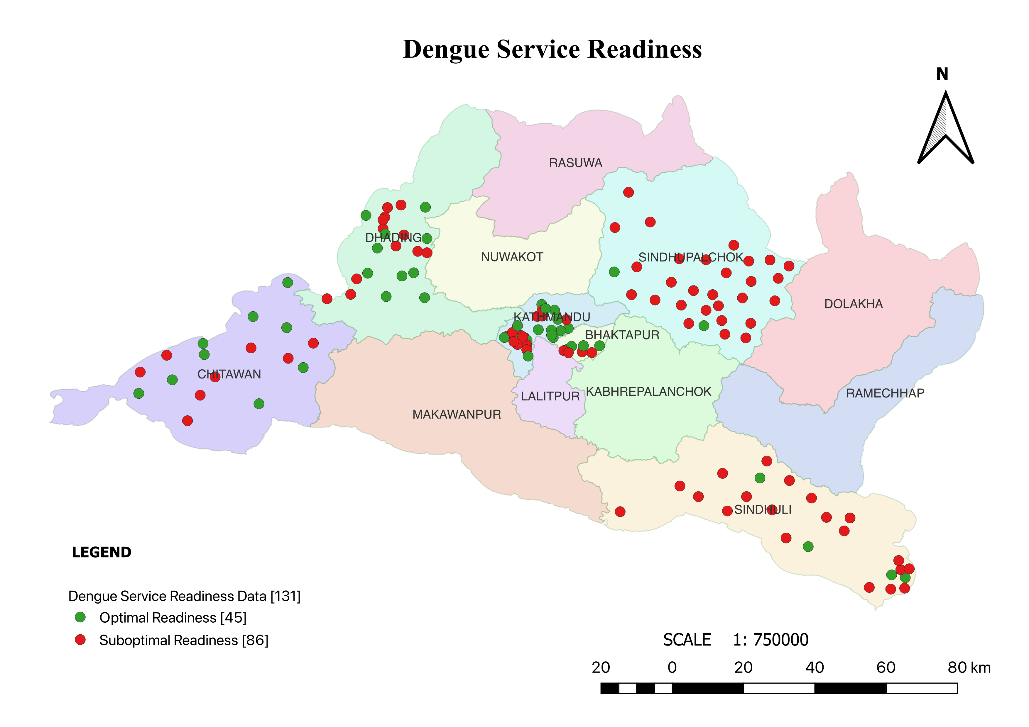


**S6: Details of Health Service Provider as key informants**

| **S.N.** | **Code** | **Health Service Provider** | **Gender** | **Service Duration** | **Facility Type** | **Ecological Region of facility** | **District of facility** | **Setting-Facility** |
| --- | --- | --- | --- | --- | --- | --- | --- | --- |
| 1. | K01 | Lab Technician | Female | 60 months | Health Post | Hill | Kathmandu | Urban |
| 2. | K02 | Facility In-Charge | Female | 33 months | PHCCs | Hill | Kathmandu | Urban |
| 3. | K03 | Health Section Chief | Male | 13 months | Municipality | Mountain | Sindhupalchok | Urban |
| 4. | K04 | Lab Technician | Male | 136 months | PHCCs | Mountain | Sindhupalchok | Urban |
| 5. | K05 | Medical Officer | Male | 23 months | Basic Hosp. | Terai | Chitwan | Rural |
| 6. | K06 | Health Section Chief | Male | 54 months | Municipality | Terai | Chitwan | Urban |
| 7. | K07 | ANM | Female | 145 months | Health Post | Hill | Dhading | Rural |
| 8. | K08 | Health Section Chief | Male | 36 months | Municipality | Hill | Dhading | Urban |
| 9. | K09 | Hospital Chief | Female | 17 months | Basic Hosp. | Hill | Bhaktapur | Urban |
| 10 | K010 | Facility In-Charge | Male | 14 months | PHCCs | Hill | Sindhuli | Urban |

**S7: Conceptual Framework of the study**

Barriers for Service Readiness

Facilitators for Service Readiness

**Disease burden & Facility Response**

- Reported cases
- Reported outbreak in district
- Review and trend analysis
- Staff assignment
- Stakeholder engagement
- ‘Search and destroy’ campaign

**Health Facility Characteristics**

- Type of health facility
- Ecological region
- Settings; urban and rural
- Staff availability status
- Distance to headquarters

**Leadership and Governance**

- Frequency of health facility meeting
- Frequency of HFOMC meetings
- External supervision in last 4 months
- Quality assurance practice
- Availability of client feedback collection mechanism

**Service Readiness for Dengue**

- Basic Amenities
- Infection prevention and control
- Diagnostic Capacity
- Essential medicines and commodities,
- Staff and guideline
